# Fluoride Modified Graded Restoratives Based on Induced Silica Mineralization

**DOI:** 10.3390/jfb17060265

**Published:** 2026-06-01

**Authors:** Ahmed K. Al-Kamal, Israa Z. Ahmed, Esraa A. Abbod, Kadhim K. Resan, Mohammed Ali Abdulrehman, Ali M. Flayyih

**Affiliations:** 1 Materials Engineering Department, Engineering College, Mustansiriyah University, Baghdad 10045, Iraq; ahmedalkamal@uomustansiriyah.edu.iq (A.K.A.-K.); edu.esraa.a@uomustansiriyah.edu.iq (E.A.A.); mohammed_ali_mat@uomustansiriyah.edu.iq (M.A.A.); ali.iq7840@uomustansiriyah.edu.iq (A.M.F.); 2 Mechanical Engineering Department, Engineering College, Mustansiriyah University, Baghdad 10045, Iraq; israazuhair@uomustansiriyah.edu.iq

**Keywords:** functionally graded materials, bioactive dental restoratives, biomimetic mineralization, hydroxyapatite, fluorapatite

## Abstract

Most existing dental restorative materials exhibit limited bioactivity, insufficient acid resistance, and poor mechanical compatibility with natural tooth structures. This study involved an in vitro approach in which a biomimetic fluoride-modified functionally gradient dental restorative material was prepared from sol–gel-derived mesoporous silica through mineralization induced via SBF solution. They synthesized bioactive restorative materials by introducing silica into a simulated body fluid (SBF) for biomimetic mineralization and generating hydroxyapatite on the silica surface. XRD, FTIR, SEM, and EDS analyses confirmed the presence of hydroxyapatite and fluorapatite-like phases. The results showed statistically significant improvements (*p* < 0.05) in the mechanical properties. The surface hardness of the developed restorative system ranged from 214 HV for the prepared silica to 392 HV for the fluoride-modified specimens. Biomimetic mineralization and fluoride modification increased the shear bond strength to dentin substrates from 9.2 MPa to 21.4 MPa and the wear from 12.8 mg to 3.6 mg, respectively. Acid resistance evaluation also showed that the specimens with fluoride modification had the highest value of hardness retention (92.1%) after acid resistance due to the formation of chemically stable and dense apatite-rich layers on the surface. The functionally graded structure demonstrated a partial biomimetic resemblance to certain hierarchical and functional characteristics of natural dental tissues under in vitro conditions. In vitro studies on bioactivity, mechanical properties, and resistance to acidic environments of the synthesized restorative showed promising results for future dental restoration applications.

## 1. Introduction

Restorative dentistry has changed dramatically over the last few decades because of the growing incidence of dental decay, erosion, and damage to tooth structures. Current dietary practices, acidic foods, and persistent bacterial films cause demineralization and degradation of the tooth surface and restorations. Although contemporary materials in restorative dentistry have been shown to be effective in replacing lost teeth, their long-term clinical performance has been restricted by insufficient bioactivity, poor resistance to acidic environments, and poor mechanical compatibility with natural teeth [1,2]. Therefore, there is a growing consensus in dental materials science that restorative dentistry should move beyond the conventional paradigm of replacement and actively engage in biological repair and functional stability.

Natural teeth have hierarchical and functionally graded structures. The enamel provides hardness, while the dentin helps provide toughness and energy dissipation. In contrast, conventional restorative materials are generally homogeneous in nature, which often results in stress concentration, debonding, and failure during cyclic mastication loading [3,4]. This incompatibility in structural architecture is one of the major limitations of conventional restorative materials, which points to the urgent requirement for materials that can mimic the functional gradation of natural teeth while being stable in the chemically aggressive oral environment.

In addition to the aforementioned structural aspects, the absence of intrinsic bioactivity in the materials used for restoration has also been found to play a major role in the development of secondary caries and marginal degradation. Therefore, bioactive materials for dental restoration, which have been found to interact with the surrounding tissues and induce the precipitation of minerals, have been developed as a new approach to replace conventional inert materials for dental restoration. Bioactive materials for dental restoration can induce the precipitation of hydroxyapatite crystals at the restoration-tooth interface, thereby improving the bonding strength between the two and the lifespan of the restoration. This marks a move towards restorative systems that can enable tissue remineralization and interfacial stability [4].

Silica-containing materials have garnered significant attention owing to their excellent biocompatibility, chemical versatility, and mineralization properties. In aqueous physiological environments, silica surfaces can readily generate silanol groups (-Si-OH) on their surfaces, which can act as catalysts for calcium and phosphate ions to precipitate HA crystals [5,6]. This process resembles the natural process of remineralization of teeth. Therefore, silica is a promising material for the development of novel dental restorative materials. However, silica-based materials are neither strong nor acid-resistant on their own. Although silica-based systems have shown promising results in terms of bioactivity, contemporary restorative systems still suffer from the inability to combine bioactivity with the desired acid resistance and mechanical behavior required for compatibility with the hierarchical structure of natural teeth. The most currently used restorative materials can be divided into two types. The first type is mechanically robust yet biologically inert, whereas the second type is biologically active but does not meet the necessary structural requirements.

To overcome these limitations, SBF-induced biomimetic mineralization has been extensively studied as a potent method to improve the bioactivity of silica-based materials. SBF, which is designed to have the ionic composition of human plasma, can be used to form calcium phosphate on the surface of materials under controlled conditions in vitro [3,4]. While past research on SBF-treated silica has focused on surface mineralization, little work has been done that integrates biomimetic mineralization, fluoride stabilization, and functionally graded architecture into one restorative system. Previous studies have reported that SBF-treated silica can improve surface bioactivity and increase resistance to acid degradation [6,7]. However, investigations of silica-based materials have mainly focused on their surface mineralization behavior, and little attention has been paid to the mechanical properties and short-term chemical stability of these materials for dental applications [6,7,8,9]. Moreover, earlier research has mainly focused on studying the effects of biomimetic apatite formation due to SBF, stabilization by fluoride ions, and graded structures in restoratives. Therefore, there is still much uncertainty about the impact of their synergistic interaction on restorative function.

In recent years, the concept of functional graded materials (FGMs) has been used to overcome the mechanical and structural shortcomings of homogeneous restoratives. FGMs can be described by spatially differentiated compositions and properties that enable custom performance in each part of a component. This method provides an attractive possibility for approximating the gradient between the enamel and dentin, thereby promoting improved stress distribution, reduced interfacial mismatch, and enhanced fracture resistance in dental applications [8,10]. Both experimental and computer-based studies have shown that graded architectures can significantly enhance the load-bearing capacity and improve the damage tolerance behavior compared to homogeneous materials [6,8]. Graded restorative approaches have proven to be more effective in terms of mechanical properties; however, there is still a lack of research on the interaction of such concepts with bioactive mineralized silicas. In particular, few studies have focused on the interaction between fluoride-stabilized apatite and gradient-based structures for enhanced resistance to both acids and stress.

While bioactive silica structures, along with the concept of functionally graded design, have achieved individual breakthroughs, little research has been conducted on their application in dental restorations. Various fluoride-containing dental restorations, namely fluoride-releasing composites, fluorapatite coatings, and glass ionomer-based materials, have been studied to increase the remineralization capabilities and acid resistance in restorative dentistry. Nevertheless, many of those restorations are homogeneous and focus more on fluoride release rather than the biomimicry of natural tooth structures. At the same time, the interaction of biomimetic silica mineralization, fluoride modification, and functionally graded structures in relation to dental restorations has never been adequately investigated. Most importantly, the interaction between fluoride-applied apatite stabilization, biomimetically induced by SBF, and the functionally graded structure within the restorative system has not been discussed at all. Among the existing biocatalytic methods, silica mineralization induced by SBF offers certain benefits owing to the possibility of forming apatite crystals using the simulated physiological environment. In addition, the silanol-rich structure of silica allows calcium phosphate precipitation and supports interfacial biocompatibility mimicking natural remineralization processes. However, such issues are hardly ever covered in any academic paper, including the interaction of laboratory conditions with clinically relevant conditions, as stated in the literature [11,12,13]. At the same time, many studies rely only on the results of in vitro tests; therefore, their accuracy cannot be determined because of the complexity of the oral environment due to changes in the pH level, among other factors [12,13,14].

In other words, addressing the above-mentioned problems will allow for the creation of materials that would demonstrate better stability and resistance in oral environments, thus increasing the effectiveness of dental restoration. The functionally graded mechanical properties of biomimetically mineralized dental restoration structures may contribute to higher structural compatibility and increased resistance to mechanical stress and corrosion. Therefore, it is important to develop a unified restorative system incorporating fluoride-applied apatite stabilization, biomimetic SBF mineralization, and functionally graded design. Furthermore, the current approach to developing materials for the present investigation is based on the concept of minimally invasive treatments, which combine biomimetic mineralization and patient healthcare practices [15,16,17,18,19,20].

In this context, the present study aims at the development of a bioactive dental restoration material, which has the potential to improve the remineralization potential, mechanical properties, and resistance of the dental structures towards the acidic environment, which might be present in the oral environment. It is a unique combination of biomimetic mineralization, fluoride treatment, and a graded restoration approach that makes this study innovative rather than its isolated utilization. In contrast to traditional homogeneous dental restorations, the gradient-inspired concept presented in this study is intended to emulate the functional architecture of biological tooth tissues, including a mineralized surface layer similar to enamel and a subsurface region similar to dentin. Simultaneously, fluorine incorporation into biomimetically formed apatite would minimize its chemical solubility, increase its crystallographic stability, and provide acid resistance. To the best of our knowledge, there have been few scientific works systematically exploring the synergistic effect of SBF-induced mineralization, fluorine treatment of apatite, and the biomimetic gradient concept of dental restoration. Therefore, the current study aimed to determine the correlation between the phase composition, microstructure formation, mechanical properties, and acid stability of the novel bioactive restorative material.

This study aimed to present a comprehensive concept for future bioactive dental restorative materials by synthesizing the concepts of biomimetics, materials science, and restorative dentistry. The results will not only lead to enhanced restorative performance but also to enhanced bioactive material design strategies that can be used in a wider biomedical context. It follows that the proposed concept is a potentially successful approach for developing biomimetic restoratives in the future that will combine bioactivity, chemical stability, and structurally motivated mechanical compatibility.

## 2. Materials and Methods

The selected materials were based on the desire to design a bioactive restoration system with a biomimetic gradient structure similar to that of natural tooth tissues. Mesoporous silica nanoparticles (MSN) (SBA-15, ≥99% pure) were purchased from Sigma-Aldrich (St. Louis, MO, USA). The other chemicals used were polyvinyl alcohol (PVA, average molecular weight 89,000–98,000, ≥99% pure) and chitosan (low-to-medium molecular weight, degree of deacetylation 75–85%), both from Sigma-Aldrich (St. Louis, MO, USA), as well as sodium fluoride (NaF, ≥99% pure) from Merck (Darmstadt, Germany). All chemicals were used as received without further purification. Silica powder was synthesized via the sol–gel method. Tetraethyl orthosilicate (TEOS) was employed as the silica precursor and underwent hydrolysis in an acidic medium (pH ≈ 2–3) in the presence of hydrochloric acid. The hydrolysis/condensation reaction mixture was constantly stirred at room temperature for several hours. Subsequently, the synthesized sol was aged at 24 °C for 24 h. The obtained gel was thoroughly rinsed with distilled water, dried at 80 °C for 24 h, and then calcinated at 600 °C for 2 h to obtain the silica powder. Thus, surface silanol groups (Si–OH) were formed. Simulated Body Fluid (SBF) was prepared according to the Kokubo protocol, which provides ionic concentrations equivalent to those of human blood plasma. Polyvinyl alcohol (PVA) and chitosan were used as the polymer-based dentin-like material, owing to their good biocompatibility. Sodium fluoride (NaF) aqueous solutions with concentrations ranging from 50 to 200 ppm were used for the post-activation treatment. Human molars removed through clinical procedures were used only as substrates for conducting adhesion studies. All teeth used in this experiment were de-identified before being used in the laboratory and were kept hydrated until the experiments began. No identifiable patient data were collected or used in this study. Based on institutional guidelines, no ethical approval was required because the teeth used in the experiment were de-identified and taken through clinical procedures only for in vitro studies. All reagents used in the current study were of analytical grade and were used as supplied.

During biomimetic mineralization, the calcined silica powder samples were immersed in SBF at 37 °C for predetermined times of 7 and 28 d. After immersion, the samples were gently washed with deionized water and then dried at room temperature. Hydroxyapatite forms on the surface of silica, mimicking the process of tooth remineralization. The sample designation and treatment conditions used for the developed silica-based restorative materials are summarized in Table 1.

To further improve the chemical stability and acidic resistance, another set of SBF-activated samples was subjected to fluoride post-treatment by immersion in a sodium fluoride solution for 6 h at room temperature. This helped in the partial substitution of hydroxyl groups in hydroxyapatite by fluoride ions to form fluorapatite-like structures.

**Figure 1:**
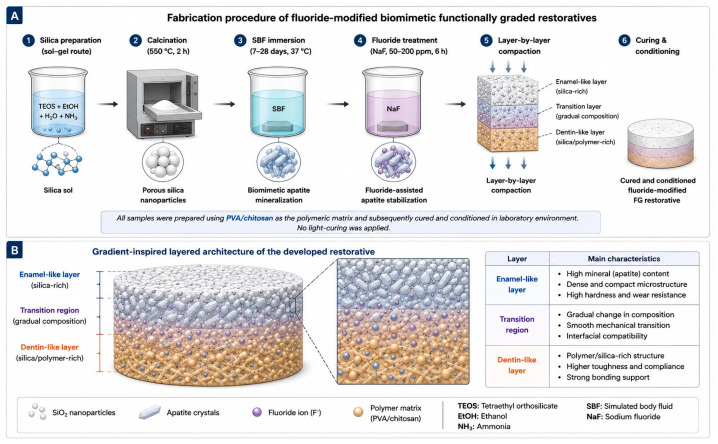
(**A**) Diagrammatic illustration of the fabrication procedure of the fluoride-modified biomimetic functionally graded restorative system, including silica synthesis, SBF-induced mineralization, fluoride treatment, and layer-by-layer compaction. (**B**) Schematic representation of the gradient-inspired layered architecture of the developed restorative material, showing the enamel-like, transition, and dentin-like regions together with their principal structural characteristics.

Functionally graded restorative specimens were designed and developed to mimic the hierarchical architecture of natural teeth in vitro. The outer layer was made of compacted SBF-activated silica powder with and without fluoride post-treatment, simulating the enamel layer, whereas the inner layer was made by mixing silica powder with a polymer matrix containing PVA and chitosan in a weight ratio of 70:30. The dimensions of the synthesized specimens were approximately 10 mm × 10 mm × 5 mm. The enamel-like external layer and the dentin-like internal layer were prepared at approximately 1.5–2 mm and 3 mm in thickness, respectively. Compaction in the layers was carried out under an applied uniaxial pressure of approximately 10 MPa to facilitate bonding between layers. After synthesis, the specimens were cured and stored under room conditions for 24 h before characterization and mechanical tests. The use of compaction in the layers ensured proper bonding between the layers, thereby achieving a gradient of properties. The gradient region between the layers was accomplished by layered compaction before solidification. The synthesized specimens were cured and conditioned in a laboratory environment before characterization and testing. A schematic representation of the layer-by-layer formation procedure and the gradation structure of the synthesized dental restoration samples is shown in Figure 1.

X-ray diffraction (XRD) was used for phase identification and mineral formation, whereas Fourier-transform infrared spectroscopy (FTIR) was used for the identification of functional groups and verification of hydroxyapatite and fluorapatite phase formation. The microstructural features and elemental composition were identified using scanning electron microscopy coupled with energy-dispersive X-ray spectroscopy (SEM/EDS), with particular emphasis on the surface morphology and Ca/P atomic ratio. XRD experiments were conducted with the help of X-ray diffractometer (INEL, Artenay, France) with the wavelength of radiation Cu-Kα (λ = 1.5406 Å) in the angular range 2θ = 10–60° with a step size of 2°/min and a voltage/current of 40 kV and 30 mA, respectively. The FTIR investigations were done using a Fourier transform infrared spectroscopy (Bruker, Billerica, MA, USA) with a spectral resolution of 4 cm^−1^ in the range of 400 to 4000 cm^−1^. SEM combined with energy-dispersive X-ray spectroscopy (EDS) analyses was done using an SEM apparatus (TESCAN, Brno, Czech Republic) was performed using an accelerating voltage of 15–20 kV in a vacuum environment to analyze the morphological and elemental structure of the prepared samples. EDS analysis was performed using an Oxford Instruments detector in conjunction with SEM.

Vickers microhardness tests were performed to determine the mechanical performance according to the standard ASTM E384 [21]. The wear resistance was tested by pin-on-disk testing following ASTM G99 [22], and the tests were carried out on the enamel-like surface of the functionally graded specimens. As the enamel-like outer layer is the one that will be primarily exposed to mechanical chewing forces and oral abrasions, it was chosen for conducting wear tests. Wear testing was performed using an abrasive material composed of stainless steel. It was performed under an applied load of 10 N, with a sliding distance of 1000 m, and at a rotation speed of 200 rpm under dry conditions. Three samples were tested for each group of experiments. The adhesion performance was assessed using the shear bond strength test per ISO 29022 [23]. The extracted human teeth were cleaned and placed in distilled water until use. The dentin surface was exposed using silicon carbide papers and then rinsed with deionized water before the bonding procedure was performed. No further dental adhesive system was applied during the bonding process to test the adhesion behavior of the new restorative material directly at the interface in vitro. Cylindrical restoration specimens were made with a diameter of approximately 4 mm in the bonded area with a mold on the dentin surface. The bonded specimens were subjected to tests using a universal testing machine at a crosshead speed of 1 mm/min.

Acid resistance was characterized by immersing specimens in a lactic acid solution (0.1 M) with a pH level of 4.5 for 72 h at 37 °C to simulate acidic environments associated with cariogenic challenges in the oral cavity. The specimens were completely submerged in the laboratory throughout the entire exposure period. All tests were conducted in triplicate to ensure reproducibility, and the results are reported as mean ± standard deviation. One-way ANOVA statistical analyses were applied to evaluate significant differences between the groups under study. Differences were considered statistically significant at *p* < 0.05.

## 3. Results and Discussion

### 3.1. Phase Formation and Microstructural Characteristics

The phase formation and microstructural evolution of the silica-based restorative material after activation in simulated body fluid were comprehensively studied. These results provide clear evidence of the progression of the transformation from an amorphous silica structure to a bioactive mineralized surface, similar to natural dental tissues.

**Figure 2:**
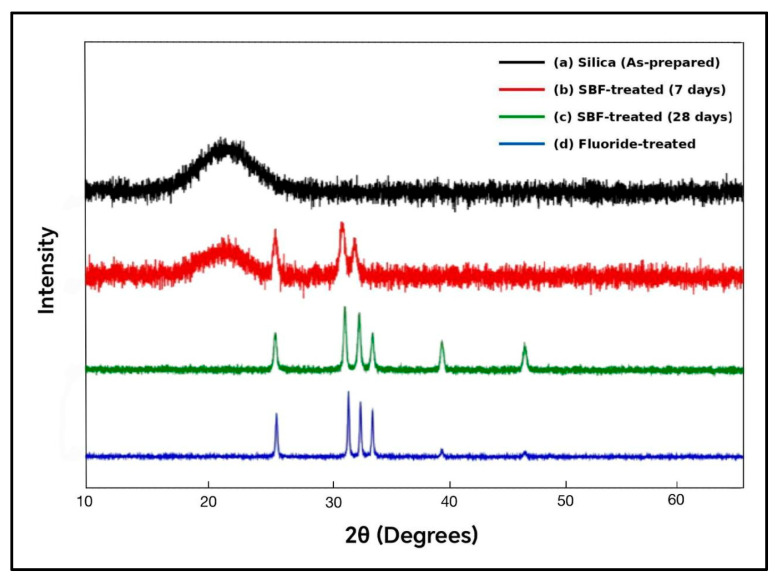
X-ray diffraction (XRD) patterns of (a) as-prepared silica, (b) SBF-treated sample after 7 days, (c) SBF-treated sample after 28 days, and (d) fluoride-treated sample. The amorphous silica shows a broad peak at 2θ ≈ 20–23°, whereas the SBF-treated samples show the characteristic peaks of hydroxyapatite at 2θ ≈ 26° and 31–33°, with increasing intensity after longer SBF treatment. The fluoride-treated sample showed improved crystallinity with slight shifts in the peaks.

The XRD patterns shown in Figure 2 reveal the characteristics of silica-based materials. The silica-based material exhibited a diffuse halo with 2θ values ranging from 20° to 23°, which is characteristic of an amorphous silica structure. The absence of crystalline phases of calcium phosphates was also confirmed. The silica surface exhibited a significant phase transformation after immersion in simulated body fluid. The appearance of diffraction peaks characteristic of hydroxyapatite was observed. The diffraction peaks found at 2θ values of 26° and 31–32° were found to correspond to the (002) and (211) crystal planes of hydroxyapatite, confirming the successful biomimetic mineralization of the silica surface. These diffraction peaks are consistent with the standard hydroxyapatite reference pattern (ICDD/JCPDS card No. 09-0432). The intensity of the diffraction peaks increased with time, ranging from 7 to 28 days, confirming the continued nucleation and growth of apatite crystallites. The absence of diffraction peaks, which are characteristic of the crystalline phases of tricalcium phosphate and calcium carbonate, was also confirmed.

In the case of the fluoride-treated sample, as shown in Figure 2, there was a slight shift in the diffraction peaks to larger angles, and the sharpness of the peaks was enhanced. This phenomenon is associated with the replacement of hydroxyl groups with fluoride ions, leading to the formation of fluorapatite-like structures. The observed peak shifting and sharpening were also consistent with the fluorapatite reference standards (ICDD/JCPDS card No. 15-0876).

**Figure 3:**
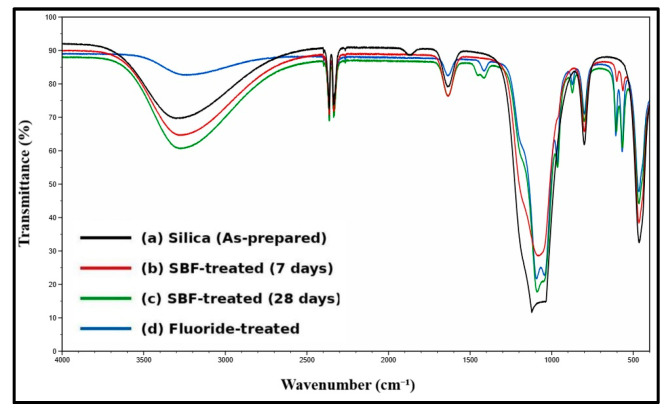
FTIR spectra of (a) as-prepared silica, (b) SBF-treated for 7 d, (c) SBF-treated for 28 d, and (d) fluoride-treated sample.

The results obtained from the FTIR spectra presented in Figure 3 confirm the formation of different phases. In the case of silica gel, the characteristic bands are associated with the stretching and bending vibrations of the silicon–oxygen–silicon bond. These bands confirmed the structure of the silica gel. In the case of silica gel treated with body fluid, new bands appeared in the range of 560–605 cm^−1^ and 1030–1090 cm^−1^, which were associated with the stretching and bending vibrations of the phosphate group. These bands confirm the formation of phases associated with calcium phosphates. The presence of hydroxyl groups indicated the formation of hydroxyapatite. In the case of fluoride-treated silica gel, there was a significant decrease in the intensity of the hydroxyl bands, which confirmed the replacement of hydroxyl groups with fluoride ions, leading to the formation of fluorapatite-like structures.

**Figure 4:**
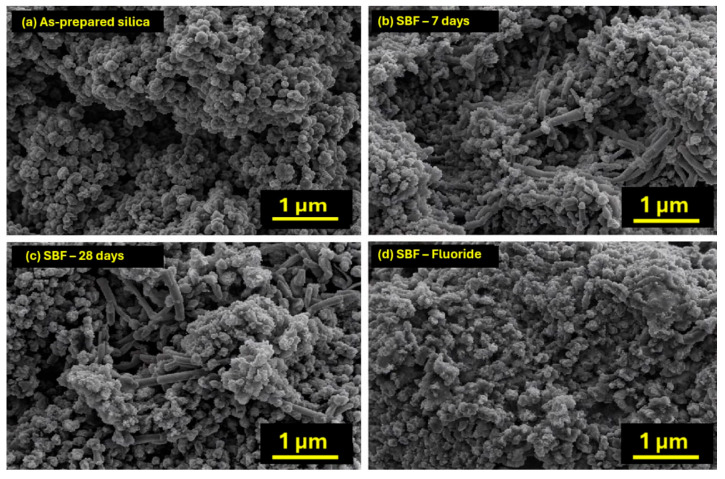
SEM images of the synthesized dental restoratives: (**a**) silica as prepared, exhibiting particle agglomeration and a porous structure; (**b**) silica after soaking in SBF for 7 days, exhibiting Ca-P nucleation and low porosity; (**c**) silica after soaking in SBF for 28 days, exhibiting dense HA deposition and rod-shaped crystals; and (**d**) fluoridated silica, exhibiting a compact fluorapatite structure.

The microstructural changes in the material are shown in Figure 4. As depicted in Figure 4a, the as-prepared silica material possessed irregular agglomerated particles with a porous and non-uniform morphology, comprising voids, boundaries of clusters, and fine particles. This morphology is attributed to the lack of mineral deposition and is indicative of a large surface area for nucleation.

As depicted in Figure 4b, after seven days of immersion in SBF, the silica surface exhibited initial biomimetic mineralization features. The presence of fine granular features and the onset of rod-like crystal growth are indicative of nucleation on the silica surface, resulting in hydroxyapatite deposition.

As depicted in Figure 4c, after 28 d of immersion in SBF, the silica surface was fully covered by a dense and continuous layer of deposited material. This morphology is indicative of nucleation and crystal growth, comprising densely packed fine granular features and rod-like crystal growth in clusters.

As shown in Figure 4d, another level of refinement in the microstructure was observed for the fluoride-treated sample. The surface contained a highly compact and interconnected network of rod-like crystals and granular apatite with low porosity. This compaction is due to the effect of fluoride ions on crystal growth and lattice ordering, leading to a stable and compact fluorapatite-rich phase.

It should be noted that the mineral layer in Figure 4b–d is seen as a surface layer covering the initial silica surface rather than forming a part of it. This is characteristic of surface-controlled biomimetic mineralization, where calcium phosphate forms on the surface and grows outwards to form a continuous layer on the top. Although classical needle-like hydroxyapatite crystals were not observed, the morphology observed is characteristic of calcium-deficient or poorly crystalline hydroxyapatite formed under biomimetic conditions, where surface-controlled nucleation prevents extensive crystal growth.

**Figure 5:**
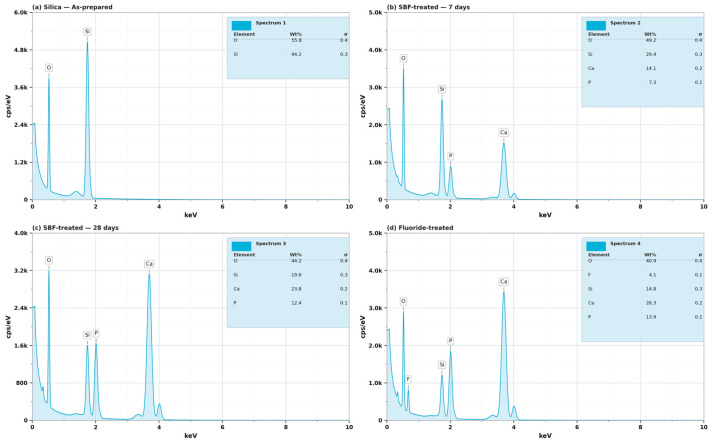
EDS spectra of (**a**) pure silica, (**b**) SBF-treated for 7 days, (**c**) SBF-treated for 28 days, and (**d**) fluorinated silica, demonstrating that the calcium and phosphorus content increases with prolonged exposure to SBF.

**Figure 6:**
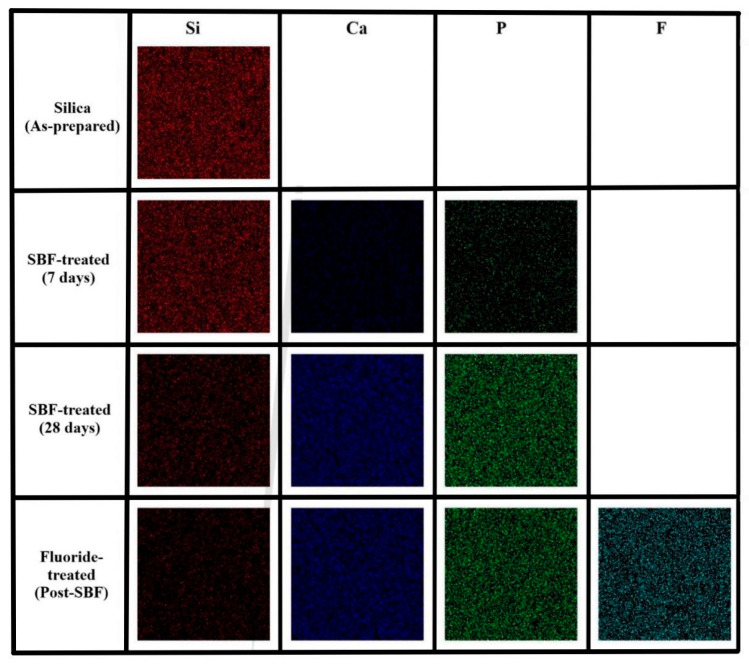
EDS elemental mapping of as-prepared, SBF-treated, and fluoride-treated samples.

Furthermore, the analysis of the EDS spectra and elemental mapping results depicted in Figure 5 and Figure 6 also validates the progression of the compositional changes caused by the biomimetic mineralization induced by SBF. Specifically, the as-prepared silica material showed only peaks related to Si and O, with no visible peaks associated with Ca, P, or F, which is consistent with the amorphous nature of the silica material. When the soaking duration increased to 7 days, peaks related to Ca and P were observed, suggesting the deposition of Ca-P compounds with a ratio of Ca/P = 1.47. Further extending the soaking period to 28 days increased the intensity of the Ca and P peaks along with a diminished peak of Si, which suggests the formation of a dense apatite layer on the silica substrate surface with a Ca/P ratio of 1.64. The addition of a fluoride-containing compound led to the appearance of F-, suggesting the presence of fluorapatite (Ca/P = 1.69).

From the evidence provided by X-ray diffraction, Fourier-transform infrared spectroscopy, scanning electron microscopy, and energy-dispersive X-ray spectroscopy, it is clear that there is a transformation from silica to a bioactive mineralized surface, with the formation of hydroxyapatite induced through exposure to simulated body fluid, while exposure to fluoride improves the stability of the structure.

The developed microstructure showed partial morphological resemblance to mineralized dental tissues under in vitro conditions, thus creating a good foundation for improved mechanical strength and resistance to acidic environments.

### 3.2. Mechanical Performance and Adhesion Behavior

The mechanical properties of the prepared restorative material were greatly affected by biomimetic mineralization in simulated body fluid, as well as the functionally graded structure. The microhardness data obtained from the Vickers hardness test, as shown in Table 2, clearly indicate a progressive improvement in the surface hardness with the degree of mineralization. The hardness tests were carried out with a test force of 500 g held for 15 s. The results presented are the average of at least three readings for each experimental case. The prepared silica had low hardness values, that is, 214 HV, at the enamel-like surface, which is consistent with the amorphous structure of the prepared material and the absence of calcium phosphate-based reinforcing phases [17]. However, the surface hardness of the prepared material increased significantly (*p* < 0.05) after biomimetic mineralization in simulated body fluid. After seven days of immersion, the surface hardness of the prepared material increased to 287 HV, which can be attributed to the formation of an initial hydroxyapatite-based surface layer. After 28 days of immersion, the surface hardness of the prepared material increased to 351 HV, which is close to the hardness of natural enamel [9]. Furthermore, the progressive decrease in hardness from the enamel-like outer layer to the transition and dentin-like layers offers an indirect indication of a gradient-induced design in the restorative material system. The enamel-like outer layer presented the highest hardness value owing to the generation of the apatite mineralization layer, whereas the inner dentin-like layer displayed a relatively lower hardness value as a result of the silica-based matrix reinforced with the polymer layer. The progressive mechanical properties of the outer and inner layers are expected to help minimize sudden changes in hardness and enhance the stress distribution when subjected to load conditions.

In contrast to homogeneous restorative materials, which usually show abrupt variations in their physical and mechanical properties at the interface, the proposed layered structure shows progressive variations in its mechanical properties throughout the thickness of the material. The intermediate hardness values recorded in the transition layer also confirm the gradient-induced design of the material rather than the brittle interface in the layers.

The highest hardness was recorded for the fluoride-treated samples, with a maximum value of 392 HV for the enamel-like surfaces. This is due to the formation of a fluorapatite-rich phase, which has higher crystallinity, better lattice stability, and lower solubility than hydroxyapatite [17]. The gradient hardening pattern of the fluoride samples was especially noteworthy, with the outer enamel-like layer being the hardest at 392 HV, followed by a decrease in the transition zone hardness to 336 HV, and a further reduction to 289 HV for the dentin-like layer. It is anticipated that this hardening gradient will assist in optimizing the load transfer and minimizing the stress concentrations. The dense and refined microstructure, as confirmed by scanning electron microscopy (SEM) (Figure 4d), further confirms that the fluoride-treated samples have better resistance to localized plastic deformation during indentation. This behavior is broadly consistent with the natural functional gradation observed between enamel and dentin tissues, where progressive changes in mechanical properties contribute to improved structural integrity and damage tolerance.

The wear resistance behavior of the outer layer of the developed material resembling the enamel layer is illustrated in Table 3. Three specimens were used for wear testing in each experimental group under similar loading conditions. The as-prepared silica has the highest wear loss, with a value of 12.8 mg, showing poor wear resistance to abrasive forces. In contrast, the samples treated in SBF showed a significant reduction in wear loss, with a value of 8.4 mg after 7 days and a further reduction to 5.1 mg after 28 days in SBF solution. This can be attributed to the formation of a hydroxyapatite layer, which acts as a barrier to prevent direct contact between the surface and silica matrix during wear tests [6,16].

The fluoride-treated samples showed the lowest wear loss, with a value of 3.6 mg, showing better wear resistance to material removal. This can be attributed to the formation of a dense and stable fluorapatite-rich phase, which has better resistance to microfractures and surface degradation [17]. The functionally graded architecture plays an important role in achieving better wear resistance, as the enamel-like surface has shown better resistance to wear, whereas the dentin-like layer has shown better resistance to stress during sliding contact, simulating the natural wear behavior of human teeth [6]. However, an SEM analysis of the surfaces exhibiting wear was not performed in this study, and should be done in further research for a better comprehension of the wear processes occurring at the surfaces.

The adhesion properties of the interface between the restorative material and the human dentin substrate were evaluated by performing a shear bond strength test. A shear bond test was performed using a universal testing machine, and the results obtained were based on an average of three specimens from each group tested at a crosshead speed of 1 mm/min. The results are presented in Table 4. The bond strength of the as-prepared silica was the least, at 9.2 MPa, due to the lack of chemical interaction and interfacial compatibility [13]. The bond strength of the silica after activation in SBF showed a statistically significant improvement (*p* < 0.05) up to 14.6 MPa after 7 days and 18.9 MPa after 28 days of SBF immersion. The improvement in bond strength is due to the formation of a bioactive calcium phosphate layer, which enhances the interfacial compatibility and adhesion behavior under in vitro conditions [4,8].

The maximum bond strength was found for the fluoride-treated specimens, which was 21.4 MPa. This value is comparable to or greater than that of many conventional restorative materials [13]. This is because of the combined effects of fluorapatite formation, surface stability, and enhanced micromechanical interlocking between the restoration and the tooth structure [20]. Unfortunately, the failure modes were not carefully analyzed in this current research and should be considered in any future research to fully understand the bonding processes at the interfaces. In addition, the slow transition between the hard and rigid external zones and the softer internal zone may be an important factor in preventing sudden stress concentrations at the interface due to shear loading. This indicates a better bond strength performance and stress distribution behavior when subjected to shear loading.

The above findings show that the synergistic effect of biomimetic mineralization and functionally graded structural design significantly improves hardness, wear resistance, and bond strength. More significantly, however, the progressive differences in hardness and mechanical properties measured along the depth of the restoration indirectly demonstrate the achievement of gradient-based design of a restorative material that can partially mimic the hierarchical mechanical properties of natural tooth structures under in vitro conditions. The additional effect of fluoride also significantly improves structural stability and reliability. These findings demonstrate that this material is effective in achieving the goal of developing a material with adequate mechanical and structural reliability and bioactivity.

### 3.3. Acid Resistance Performance

The acid resistance behavior of the developed restorative materials was evaluated using the surface hardness retention test after exposure to an acidic environment. The results presented in Table 5 indicate that the acid resistance of the developed materials is dependent on the extent of biomimetic mineralization and the presence of fluoride in the surface layer.

The as-prepared silica sample exhibited the poorest acid resistance among the tested materials, as indicated by a significant decrease in the hardness value from 214 to 142 HV after 72 h in an acidic solution, resulting in a hardness retention value of 66.4%. The degradation of the silica sample was due to the amorphous structure of the silica material and the lack of a protective mineral layer, which enabled the acidic solution to interact directly with the material surface and degrade the material [14].

In contrast, the SBF-treated samples showed higher resistance to degradation in acidic solutions than the as-prepared silica sample. The 7-day SBF-treated sample showed a hardness retention value of 80.8%, as indicated by the decrease in the hardness value from 287 to 232 HV after exposure to the acidic solution. The higher resistance of the SBF-treated sample is due to the formation of the first stage of the hydroxyapatite mineral layer, which does not fully cover the material surface and thus enables the acidic solution to degrade the material [17].

Further improvement in acid resistance was noted for the sample treated for 28 days, which retained 86.9% of its original hardness, that is, from 351 HV to 305 HV. This improved hardness retention can be attributed to the development of a thicker hydroxyapatite layer, as confirmed by X-ray diffraction and scanning electron microscopy analyses.

The highest acid resistance of the fluoride-treated sample was also noted, retaining 92.1% of its original hardness, that is, from 392 to 361 HV. The chemical stability of the sample can be attributed to the development of fluorapatite-rich phases, which have lower solubility, higher ionic bonding, and higher thermodynamic stability than hydroxyapatite [17]. Moreover, the compact microstructure of the sample, as confirmed by the scanning electron microscopy micrographs, also improved the acid resistance behavior of the sample. An enhanced capacity for short-term acid resistance may become especially pertinent in cariogenic dental environments when restorative materials are subjected to acidic conditions due to bacterial fermentation, as well as consumption of acidic foods.

The hardness retention of the sample was thus directly correlated with the progressive transformation of the sample composition, as confirmed by X-ray diffraction, Fourier-transform infrared spectroscopy, and energy-dispersive X-ray spectroscopy analyses. The progressive transformation from amorphous silica to hydroxyapatite and finally to fluorapatite results in reduced chemical solubility of the sample, thereby improving its structural integrity [4,8].

In this regard, the results of the current study reveal that the higher level of acid resistance is due to the denser microstructure and better compositional stability of the fabricated restorative material. The combination of both SBF-induced biomimetic mineralization and fluoride was responsible for the formation of an acid-resistant surface with chemical stability similar to that of natural enamel. Therefore, the fabricated functionally graded restorative material exhibited higher levels of chemical stability and acid resistance in vitro.

### 3.4. Integrated Performance Assessment and Potential Restorative Implications

Conclusions from the integration of the experiments indicated that the synergy of the interactions of biomimetic mineralization, fluoride-mediated stabilization of apatite, and gradient-based architecture design was crucial in improving the mechanical strength, acid resistance, and interface properties of the fabricated restorative system. The results of this study clearly show that bioactivity, mechanical integrity, and short-term chemical stability under acidic conditions, which are essential for the development of advanced dental restorative applications, can be attained simultaneously when simulated body fluid-activated silica is incorporated into a graded structural design [1,4].

In terms of structure, the presence of a hard layer containing apatite was instrumental in ensuring that an enamel-like layer with higher strength against mechanical and acidic damage was achieved. In contrast, the relatively softer dentin-like layer acted as an energy-absorbing layer that assisted in stress dissipation. This gradual transition from one layer to another reduces any abrupt change in the mechanical properties. This hierarchy of organization partially resembles the structural organization of natural teeth under in vitro conditions, in which gradual changes in mechanical properties are necessary to reduce the concentration of stress and catastrophic fractures when stress cycles are applied [4,7]. The lack of sharp interfaces in the graded structure also contributes to structural stability.

Based on the mechanical approach, the progressive increase in hardness, abrasion resistance, and interfacial bonding strength achieved by SBF and fluoride stimulation represents the mechanical strengthening of the mineralized surface. This was attributed to the formation of increasingly dense and crystalline apatite structures, according to the results obtained from XRD, FTIR, and SEM examinations. The maintenance of mechanical strength in an acidic environment further signifies the structural stability of the mineralized surface stabilized with fluoride ions [6,17].

The increase in acid resistance in the fluorapatite-containing specimens could be attributed to decreased solubility and lattice stability, which reduced the rate of ion dissolution and surface corrosion. Moreover, it can be inferred that the dense microstructure helps prevent acidity from penetrating the surface of the material, thus protecting the surface from corrosion during long-term exposure to acidic conditions. These factors contribute to stability in areas where pH levels vary, such as the mouth [12,14].

Adhesive bond strength to dentin substrates is highly relevant in restorative applications, as it signifies better compatibility and micromechanical interactions between the adhesive layer and natural tooth under in vitro conditions. Moreover, its bioactivity, as evidenced by its potential to induce apatite formation, suggests that this material demonstrated the ability to support apatite formation under in vitro conditions compared with conventional inert restorative materials [8].

In general, the synthesized restorative system showed superior properties in comparison with traditional restorative materials, which depended mainly on mechanical strength rather than bioactivity. The combination of the biomimetic formation of mineral phases, fluoride-based apatite stabilization, and gradient-oriented structure allowed for concurrent improvements in mechanical compatibility, acid resistance, and interfacial properties. In addition, the mechanical gradient across the depth of the synthesized restorative material represents indirect proof of the successful fabrication of the gradient-oriented architecture, which provides a partial reproduction of the natural hierarchy of dental tissues in terms of mechanical strength. Therefore, it is evident that the interaction between mineralization, mechanical gradient, and fluoride-mediated structure stabilization renders the synthesized material a prospective dental biomimetic restorative system.

## 4. Conclusions

An innovative biomimetic fluoride-doped functionally graded dental restorative system was successfully fabricated using the concept of biomimetic mineralization initiated by an SBF solution. The synergistic role of biomimetic mineralization, fluoride-mediated stabilization of apatitic minerals, and gradient-induced organization enhances the mechanical robustness, acid resistance, and interfacial integration of the synthesized restorative system. X-ray diffraction, Fourier-transform infrared spectroscopy, scanning electron microscopy, and energy-dispersive spectroscopy analyses indicated that hydroxyapatite was formed in the SBF solution-treated samples, whereas fluorapatite-like compounds were detected after fluoride doping. The highest microhardness value, minimum wear volume, and acid degradation resistance were attributed to the formation of a tightly packed mineralized coating layer in the fluoride-doped specimens. In addition, significant improvements in the shear bond strength to dentin substrates were observed after biomimetic mineralization and fluoride treatment. The gradual change in hardness from enamel-like to transition to dentin-like layers also served as an indirect indicator of the successful fabrication of a mechanically compatible gradient-inspired restorative structure with the potential capability of simulating the hierarchical properties of biological dental structures. Although the fabricated restorative material exhibited encouraging results for in vitro biocompatibility, mechanical properties, and short-term acid resistance, the current study did not explore long-term intraoral behavior attributes, such as fatigue properties, microleakage resistance, thermal aging, and long-term biocompatibility. Hence, further long-term in vitro and in vivo studies are necessary before its clinical application. Overall, the produced restorative system showed promising results in terms of bioactivity, mechanical behavior, and acid stability. Despite the promising bioactivity, mechanical properties, and short-term acid resistance of the designed restorative material, additional aging, thermal cycling, fatigue, biological studies, and in vivo testing are necessary prior to clinical application.

## Figures and Tables

**Table 1:** Sample designation and treatment conditions of the developed silica-based restorative material, including simulated body fluid immersion time and fluoride post-treatment.

Sample ID	Description	SBF Immersion Time (Days)	Fluoride Treatment	Expected Phase
A	As-prepared silica	0	No	Amorphous SiO_2_
B	SBF-treated	7	No	Early hydroxyapatite
C	SBF-treated	28	No	Developed hydroxyapatite
D	Fluoride-treated (post-SBF)	28	Yes	Fluorapatite-like phase

**Table 2:** The Vickers microhardness values of the developed restorative materials were measured across the enamel-like, transition, and dentin-like regions.

Sample	Enamel-Like (HV)	Transition (HV)	Dentin-Like (HV)
(a) Silica (As-prepared)	214 ± 3.2	178 ± 2.8	152 ± 2.5
(b) SBF-treated (7 days)	287 ± 4.1	243 ± 3.6	201 ± 3.1
(c) SBF-treated (28 days)	351 ± 5.3	298 ± 4.2	247 ± 3.8
(d) Fluoride-treated	392 ± 6.0	336 ± 4.9	289 ± 4.3

**Table 3:** Wear loss of the developed restorative materials under sliding conditions.

Sample	Wear Loss (mg)
(a) Silica (As-prepared)	12.8 ± 0.6
(b) SBF-treated (7 days)	8.4 ± 0.5
(c) SBF-treated (28 days)	5.1 ± 0.4
(d) Fluoride-treated	3.6 ± 0.3

**Table 4:** Shear bond strength between the developed restorative materials and dentin substrate.

Sample	Shear Bond Strength (MPa)
(a) Silica (As-prepared)	9.2 ± 0.7
(b) SBF-treated (7 days)	14.6 ± 0.9
(c) SBF-treated (28 days)	18.9 ± 1.1
(d) Fluoride-treated	21.4 ± 1.3

**Table 5:** Surface hardness before and after acid exposure and the corresponding hardness retention of the developed restorative materials.

Sample	Initial Hardness (HV)	After Acid (HV)	Hardness Retention (%)
(a) Silica (As-prepared)	214 ± 3.2	142 ± 2.9	66.4
(b) SBF-treated (7 days)	287 ± 4.1	232 ± 3.7	80.8
(c) SBF-treated (28 days)	351 ± 5.3	305 ± 4.6	86.9
(d) Fluoride-treated	392 ± 6.0	361 ± 5.2	92.1

## Data Availability

Data are available from the corresponding author upon reasonable request.

## Abbreviations

The following abbreviations are used in this manuscript:

MSN	Mesoporous Silica Nanoparticles
SBA-15	Santa Barbara Amorphous-15 (type of mesoporous silica)
SBF	Simulated Body Fluid
PVA	Polyvinyl Alcohol
NaF	Sodium Fluoride
HAp/HA	Hydroxyapatite
Si-OH	Silanol groups
CaPO_4_	Calcium Orthophosphate
SiO_2_	Silicon Dioxide
XRD	X-Ray Diffraction
FTIR	Fourier-Transform Infrared Spectroscopy
SEM	Scanning Electron Microscopy
EDS	Energy-Dispersive X-ray Spectroscopy
HV	Vickers Hardness
ASTM	American Society for Testing and Materials
ISO	International Organization for Standardization
FGM	Functionally Graded Materials

## References

[1] Alshahrani M.T., Rubehan R.A., Alabduljabbar R.M. (2025). Bioactive restorative materials: The intersection of restoration and regeneration. J. Healthc. Sci..

[2] Sivamani S., Anitha V., Fatima N., Ramani S. (2023). Dental materials: A comprehensive review of evolution, classification, challenges and future prospects. Int. J. Curr. Res. Rev..

[3] Hameed R.M. (2023). Bioactive Materials Used in Pediatric Dentistry. Bachelor’s Thesis.

[4] Zafar M.S., Amin F., Fareed M.A., Ghabbani H., Riaz S., Khurshid Z., Kumar N. (2020). Biomimetic aspects of restorative dentistry biomaterials. Biomimetics.

[5] Perez-Moreno A., Reyes-Peces M.d.l.V., de los Santos D.M., Pinaglia-Tobaruela G., de la Orden E., Vilches-Pérez J.I., Salido M., Piñero M., de la Rosa-Fox N. (2020). Hydroxyl Groups Induce Bioactivity in Silica/Chitosan Aerogels Designed for Bone Tissue Engineering. In Vitro Model for the Assessment of Osteoblasts Behavior. Polymers.

[6] Dulger K., Purcek G. (2025). A comprehensive review of wear in restorative dentistry: From enamel to advanced restorative materials. Tribol. Ind..

[7] Woźniak-Budych M.J., Staszak M., Staszak K. (2023). A critical review of dental biomaterials with an emphasis on biocompatibility. Dent. Med. Probl..

[8] Paryani M., Bhojwani P.R., Ikhar A., Reche A., Paul P. (2023). Evolution of biomimetic approaches for regenerative and restorative dentistry. Cureus.

[9] Lile I.E., Cojocariu C., Pasca C., Stăncioiu A.-A., Vaida L.L., Marian D. (2026). Flexural Strength of Different Restorative Materials Used for Direct Restoration in Pediatric Dentistry: An In Vitro Study. Biomimetics.

[10] Peyton F.A. (1968). Materials in restorative dentistry. Ann. N. Y. Acad. Sci..

[11] Vermudt A., Kanis L.A., Pereira J.R. (2024). pH change of different dental adhesives 30 and 60 days after polymerization. J. Res. Dent..

[12] Satou R., Sugihara N. (2024). In vitro risk assessment of dental acid erosion caused by long-term exposure to oral liquid bandages. Dent. J..

[13] Alshehri Z.A., Azouz F.A., Almutairi A.T., Al-Johani M.H., Alanazi R.O., Almakrami A.M. (2024). Evaluation of longevity and performance of various dental filling materials. J. Healthc. Sci..

[14] Buzatu B.L.R., Buzatu R., Galuscan A., Luca D.A., Rosianu R.S., Balean O., Vlase T., Jumanca D. (2024). Assessing the impact of acidic drinks on the structural integrity of enamel and their consequences for dental aesthetics. Rom. J. Oral Rehabil..

[15] Selvaraj M., Mohaideen K., Sennimalai K., Gothankar G.S., Arora G. (2023). Effect of oral environment on contemporary orthodontic materials and its clinical implications. J. Orthod. Sci..

[16] Saha S., Roy S. (2023). Metallic dental implants wear mechanisms, materials, and manufacturing processes: A literature review. Materials.

[17] Dorozhkin S.V. (2022). Calcium orthophosphate (CaPO_4_)-based bioceramics: Preparation, properties, and applications. Coatings.

[18] Duncan H.F. (2022). Present status and future directions—Vital pulp treatment and pulp preservation strategies. Int. Endod. J..

[19] Kim J.C., Lee M., Yeo I.-S.L. (2022). Three interfaces of the dental implant system and their clinical effects on hard and soft tissues. Mater. Horiz..

[20] Eftekhar Ashtiani R., Alam M., Tavakolizadeh S., Abbasi K. (2021). The role of biomaterials and biocompatible materials in implant-supported dental prosthesis. Evid.-Based Complement. Altern. Med..

[21] (2022). Standard Test Method for Microindentation Hardness of Materials.

[22] (2023). Standard Test Method for Wear Testing with a Pin-on-Disk Apparatus.

[23] (2013). Dentistry—Adhesion—Notched-Edge Shear Bond Strength Test.

